# Anti-inflammatory Effect of Astragalin and Chlorogenic Acid on *Escherichia coli*-Induced Inflammation of Sheep Endometrial Epithelium Cells

**DOI:** 10.3389/fvets.2020.00201

**Published:** 2020-04-30

**Authors:** Xuequan Hu, Meng Wang, Yangyang Pan, Yingying Xie, Jinhui Han, Xingyun Zhang, Robert Niayale, Honghong He, Qin Li, Tian Zhao, Yan Cui, Sijiu Yu

**Affiliations:** ^1^College of Veterinary Medicine, Gansu Agricultural University, Lanzhou, China; ^2^Technology and Research Center of Gansu Province for Embryonic Engineering of Bovine and Sheep & Goat, Lanzhou, China; ^3^Lanzhou Institute of Husbandry and Pharmaceutical Sciences, Chinese Academy of Agricultural Sciences, Lanzhou, China

**Keywords:** astragalin, chlorogenic acid, *E. coli*, endometritis, TLR4/NF-κB

## Abstract

Endometritis is one of the main diseases which harm sheep husbandry. Astragalin and chlorogenic acid (CGA) are common active ingredients of traditional Chinese medicine (TCM) with immunoprotective, antioxidant, and anti-inflammatory properties. In the present study, sheep endometrial epithelium cells (SEECs) were successfully purified and identified, and the *in vitro* inflammation model of SEECs induced by *Escherichia coli (E. coli)* was successfully established. To explore the effect of astragalin and CGA on the inflammation induced by *E. coli* and its potential mechanism, six groups were set up, namely, group C, M, astragalin, CGA, BAY, and STR. Cells in group C were incubated with DMEM/F12 for 6 h, while cells in group M, astragalin, CGA, BAY, and STR were incubated with DMEM/F12, astragalin, CGA, BAY, and STR for 3 h, respectively, followed by *E. coli* infection at a multiplicity of infection (MOI) of 1 *E. coli* per cell for 3 h. Subsequently, the cells and the supernatant were collected to detect the expression of genes in the toll-like receptor 4 (TLR4)/nuclear factor-kappa B (NF-κB) pathway by ELISA, qPCR, and western blot. The results showed that *E. coli* could induce inflammation of SEECs *in vitro*, while astragalin and CGA could alleviate the inflammatory response induced by *E. coli* via inhibiting the activation of the TLR4/NF-κB signaling pathway, which provides a theoretical and experimental foundation for preventing sheep endometritis clinically.

## Introduction

With the rapid development of the Chinese economy and the improvement of living standards, the breeding industry gets more and more attention including sheep husbandry. However, the perinatal disease of sheep, especially endometritis, restricts the development of sheep husbandry, which results in a substantial economic loss (1). However, not all endometritis can cause injury to the organism; for instance, endometritis induced by breeding is a normal physiological reaction in the mare, and it is a necessary inflammatory response for the effective removal of contaminating bacteria and excess spermatozoa introduced into the uterus. Furthermore, in most cases, the endometritis resolves within 24–48 h, leaving the uterus clean and free of inflammation in time for the arrival of the conceptus as it migrates from the oviduct (2). But many female animals fail to resolve the endometritis and persistent endometritis results, which can cause sub-involution of uterus, reduced milk production, fertilization failure, infertility, and even systemic disease (3, 4). Persistent endometritis results from a response to the introduction of air, urine, semen, bacteria, fungus, and yeast into the uterus (2), with the leading cause being bacterial infection, particularly *E. coli, Staphylococcus aureus*, and *Streptococcus agalactiae* (5, 6). Lipopolysaccharide (LPS), a component of the cell wall in gram-negative bacteria, such as *E. coli*, could activate the toll-like receptor 4 (TLR4)/nuclear factor-kappa B (NF-κB) signaling pathway and lead to a strong inflammatory response. It will result in great damages to the organism if the inflammatory response persists (7). TLR4 mediates several processes in the inflammatory cascade, triggering inflammation through myeloid differentiation primary response gene 88 (MyD88)-dependent and MyD88-independent pathways. TLR4, which was activated by LPS, activates I kappa B kinase (IKKs) via a series of biochemical reactions, which degrades NF-κB inhibitory protein inhibitor kappa B (IκB). Afterward, NF-κB translocates into the nucleus and activates the expression of inflammatory genes (8, 9).

Astragalin (Supplementary Figure 1A) is a flavonoid isolated from persimmon leaves and green tea seeds which has been used for treating many diseases in traditional Chinese medicine (TCM) for many years (10). It has been demonstrated that astragalin could suppress the proliferation of hepatocellular carcinoma cells both *in vitro* and *in vivo*, and this result is associated with inhibition of hexokinase 2 through the upregulation of miR-125b and subsequent metabolic reprogramming (11). Astragalin could also alleviate IL-1β-induced inflammatory mediators via activating the peroxisome proliferator-activated receptor (PPAR)-γ, which subsequently inhibited IL-1β-induced NF-κB and mitogen-activated protein kinase (MAPK) activation (12). Chlorogenic acid (CGA) (Supplementary Figure 1B), a phenolic compound that has been identified in coffee, fruits, and vegetables, exerts antioxidant (13), anti-inflammatory (14), anticarcinogenic (15), and antibacterial (16) activities. Gao et al. reported that CGA could be used as a potential therapeutic compound for bovine mastitis due to its anti-inflammatory role by inhibiting NF-κB activation (17). However, there is no evidence of whether astragalin and CGA can alleviate the inflammatory response of endometritis induced by *E. coli* infection in sheep. Therefore, the present study aimed at creating an *in vitro* inflammation model of SEECs and evaluating the anti-inflammatory effect and potential mechanism of astragalin and CGA on *E. coli*-induced inflammation of SEECs.

## Materials and Methods

### Purification and Identification of SEECs

Sheep uteri were obtained from a slaughterhouse in Zhonghua, Lanzhou, and brought to the lab with 37°C normal saline in 2 h. Endometrium from the cornua of the uterus was collected, which was shredded to a particle size of 1 mm^3^, followed by digestion with 0.1% collagenase for 3–6 h (18). A 0.15-mm cell strainer was used to remove undigested cell mass, followed by centrifuging at 1,000 rpm for 10 min. The cells were cultured with DMEM/F12 with 20% FBS, and the differential digestion method was used to purify the epithelial cells.

Cytokeratin 18 (CK18) is a characteristic protein of epithelial cells, so the immunofluorescence assay was conducted to detect the expression of CK18 for cell identification. Cells were planted into 24-well plates (4 × 10^5^ cells per well) and were cultured in an incubator at 37°C overnight. The cells were fixed with 4% paraformaldehyde for 30 min and washed twice with PBS, followed by permeabilization with 0.5% Triton X-100 for 10 min and blocking with QuickBlock™ Blocking Buffer for Immunol Staining (Beyotime, Beijing, China) for 30 min. The cells were stained with cytokeratin 18 (1:200) overnight at 4°C, and a secondary antibody against mouse IgG (1:400) was added for 1 h at room temperature in the dark. PBS was used to replace CK18 in the negative control, and the rest remains unchanged. The cells were then stained with DAPI (Coolaber, Beijing, China) for 3 min in the dark and then photographed using a fluorescence inverted microscope (Olympus, Japan).

### Growth Study of SEECs

SEECs were seeded in 96-well culture plates at a density of 1 × 10^3^, 5 × 10^3^, 1 × 10^4^, 5 × 10^4^, 1 × 10^5^, 2.5 × 10^5^, and 5 × 10^5^ cells per well and cultured for 1 h when the cells grew adherently to the wall. A 10-μl cell counting kit-8 (CCK-8) reagent (Solarbio, Beijing, China) was added and incubated at 37°C for 1.5 h. The optical density (OD) was measured at 450 nm using a microplate reader (Bio-Rad, USA). A standard curve was made with OD and cell quantity/well as ordinate and abscissa, respectively. Meanwhile, SEECs were seeded in 96-well culture plates at a density of 10^3^ cells per well and cultured for 1 h when the cells grew adherently to the wall. Ten microliters of CCK-8 reagent was added into five wells and incubated at 37°C for 1.5 h. This process was repeated every 24 h. The OD was measured at 450 nm using a microplate reader. After that, the growth curve was made with OD and cell quantity/well as ordinate and abscissa, respectively.

### Cytotoxicity Assay

The astragalin and CGA (Yuanye, Shanghai, China) were dissolved with dimethyl sulfoxide (DMSO) and diluted with DMEM/F12 (astragalin was diluted to 400, 200, 100 50, 25, and 12.5 μg/ml, and CGA was diluted to 200, 100, 50, 25, 12.5, and 6.25 μg/ml), respectively. BAY 11-7085 (MCE, USA), the inhibitor of NF-κB activation and phosphorylation of IκBα, was also dissolved with DMSO and diluted with DMEM/F12 to 200, 100, 50, 20, 10, 5, 1, 0.5, 0.25, and 0.125 μM. SEECs were seeded in 96-well culture plates at a density of 5 × 10^4^ cells per well and cultured for 24 h. Cells were then exposed to a series of concentrations of astragalin, GA, and BAY at 37°C for 24 h. A 10-μl CCK-8 reagent was added and incubated at 37°C for 1.5 h. The OD was measured at 450 nm.

### Selection of *E. coli* MOI

*Escherichia coli* (ATCC25922) was inoculated in 10-ml LB liquid medium for 18 h at 37°C with shaking at a speed of 180 rpm. SEECs were seeded in six-well culture plates at a density of 4 × 10^5^ cells per well and cultured for 24 h. Cells were then exposed to *E. coli* at a series of multiplicities of infection (MOIs) (1:10, 1:1, 5:1, 10:1, and 100:1) and incubated at 37°C for 3 h. Changes of cellular morphology of SEECs in each well were analyzed, and apoptosis and necrocytosis of SEECs were tested using YF-Annexin V and PI Apoptosis Kit (US Everbright Inc., Suzhou, China). Total RNA was isolated, and qPCR was performed to detect the expression of NF-κB, IL-1β, and IL-6. The result was a selection criterion of the MOI of *E. coli*.

### Cell Grouping and Treatment

To evaluate the protective effect of astragalin and CGA on *E. coli* infection-induced inflammation of SEECs, six groups were set up, including groups C, M, astragalin, CGA, BAY, and STR. Cells in group C were incubated with DMEM/F12 for 6 h, while cells in groups M, astragalin, CGA, BAY, and STR were incubated with DMEM/F12, astragalin, CGA, BAY, and STR for 3 h, respectively, followed by *E. coli* infection at an MOI of one *E. coli* per cell for 3 h in the preexisting medium. Subsequently, the cells and the supernatant were collected to detect the expression of genes in the TLR4/NF-κB pathway by ELISA, qPCR, and western blot. Each *in vitro* experiment was repeated three times.

### QPCR Analysis

The total RNA was isolated from the cells with a TRIpure reagent (Bioteke, Beijing, China) according to the manufacturer's guide. Briefly, the cells were treated with RL lysis buffer and chloroform. After the removal of protein, the total RNA was dissolved in RNase-free water. The concentration of the total RNA was determined using a UV-Vis spectrophotometer Q5000 (Quawell, USA) at 260/280 nm. The first-strand complementary DNA (cDNA) was synthesized from 450 ng of total RNA using the PrimeScript™ RT reagent kit with gDNA Eraser (Takara, Japan). The synthesized cDNA was stored at −80°C until ready for use. qPCR was used to detect gene expression of TLR4, IKKβ, NF-κB, IL-1β, IL-6, IL-8, IL-12α, and TNF-α with the Light Cycler 96 System (Roche, Basel, Switzerland). Reaction mixtures consisted of the following: 10 μl of 2 × TB green premix (Takara, Japan), 0.8 μl of each primer (10 μM), 2 μl of cDNA, and 6.4 μl of RNase-free water. The PCR program for the eight genes and β-actin was one cycle at 95°C for 30 s, 40 cycles at 95°C for 5 s, annealing at 60°C for 30 s, and extension 72°C for 30 s. The primers used in the present study were designed with Primer Premier 5 software, synthesized by Sangon Biotech (Shanghai, China) and are listed in Table 1. The relative mRNA levels were calculated according to the 2^−ΔΔCt^ method accounting for gene-specific efficiencies and were normalized to the mean expression of the reference gene β-actin.

**Table 1:** Gene-special primers used for qPCR.

**Gene**	**Primer sequence**	**Gene ID**
TR4	GGTTTCCACAAGAGCCGTAA	554263
	CTGTTCAGAAGGCGATAGA	
IKKβ	TGCCGAGAAGAGTGACGACCTG	780484
	GCTGTTCCTCCTCCTCCGACTG	
NF-κB	CACCTTCTCCCAGCCCTTTG	101113005
	TGCCACCTCCTCCTCCAG	
IL-1β	ATGGGACTTCTGTGGTGTGT	443539
	TGAATGGGCTGTGCTGTAGT	
IL-6	ACACTGACATGCTGGAGAAGATGC	443406
	CCGAATAGCTCTCAGGCTGAACTG	
IL-8	GCTGGCTGTTGCTGTCTTGG	443418
	GGGTGGAAAGGTGTGGAATGTG	
IL12-α	TTCAGAATTCGTGCGGTGAC	443064
	CCCTCCCCAGTTCTTAACCC	
TNF-α	TCTGGGCAGGTCTACTTTGG	443540
	AGTCCTTGGTGATGGTTGGT	
β-actin	CCTCACGGAACGTGGTTACA	443340
	CAGTAGCCATCTCCTGCTCG	

### Western Blot Assay

Total protein was extracted with RIPA buffer (high) with PMSF and protein phosphatase inhibitor (all-in-one, 100×) (Solarbio, China). Proteins extracted were boiled for 10 min with loading buffer (Solarbio, China) and subjected to 10% SDS-PAGE under reducing conditions. The separated proteins were then transferred to PVDF membranes for 45 min (β-actin, pIκB, and IκB) or 90 min (TLR4, pNF-κB, and NF-κB) at 200 mA in a transfer apparatus (Bio-Rad, Philadelphia, USA). Membranes were blocked with 5% Bovine Serum Albumin (BSA) for 2 h at room temperature and incubated overnight at 4°C with diluted primary antibodies against TLR4 (1:1,000, Bioss, Beijing, China), IκB (1:1,000, CST), pIκB (1:1,000, CST), NF-κB (1:1,000, CST), and pNF-κB (1:500, Bioss) separately. The samples were then incubated with horseradish peroxidase (HRP)-conjugated secondary antibody against rabbit/mouse IgG (1:1,000, Bioss) for 40 min at room temperature. To verify equal loading of the samples, the membrane was incubated with a monoclonal β-actin antibody (1:1,000, Bioss,), followed by an HRP-conjugated goat anti-mouse IgG (1:1,000, Bioss) secondary antibody. The signal was detected with Amersham Imager 600 (General Electric Company, USA) and quantified by densitometry using ImageJ software. Subsequently, the densitometry of TLR4, pIκB, and pNF-κB was normalized to β-actin, IκB, and NF-κB, respectively.

### ELISA

The protein levels of TLR4 in cells and IL-1β, IL-6, IL-8, and TNF-α in the cell supernatant were measured by ELISA kits (Jingkang, Shanghai, China) according to the manufacturer's instructions. Briefly, when intracellular proteins were detected, cells were digested and made into cell suspension with PBS, followed by multigelation and centrifuging to obtain the supernatant. And when secretory proteins were detected, the culture supernatant was collected. Ten microliters of supernatant and 40-μl sample dilution were added into the 96-well plate, followed by addition of 100-μl HRP-conjugated reagent to each well and incubation at 37°C for 60 min. Fifty microliters of Chromogen Solution A and 50 μl of Chromogen Solution B were added to each well and incubated at 37°C for 15 min after washing the plate five times. Finally, 50 μl of stop solution was added, and the absorbance was detected at 450 nm with a microplate reader (Bio-Rad, USA). These steps follow the manufacturer's instructions strictly.

### Statistical Analysis

All statistical analyses were performed using the Statistical Package for the Social Sciences (SPSS) 17.0 software package, and all data were assessed using the one-way analysis of variance (ANOVA). The figures and graphs were prepared with GraphPad Prism version 7.0 for Windows. The differences of one group from the others were considered to be significant when *P* < 0.05.

## Results

### Purification and Identification of SEECs

**Figure 1:**
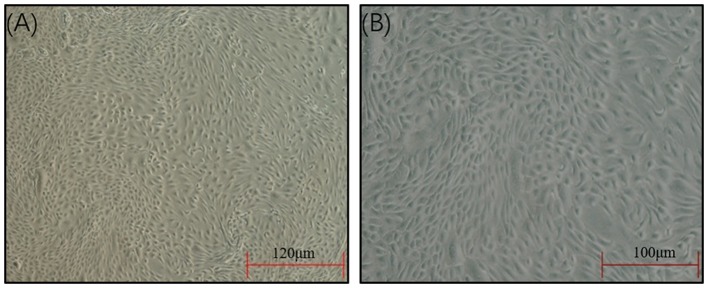
Morphology of the SEECs after purification. **(A)** The SEECs were well attached and grew, appearing as oval shapes or pebble shapes. **(B)** The SEECs were plump and rounded and closely arranged with clear boundaries.

The SEECs were successfully isolated and purified with the methods of collagenase and differential digestion, respectively. The passaged cells (Figure 1) were well attached and grew, appearing as oval shapes or pebble shapes with clear boundaries. The cells were further passaged up to the ninth generation, and there was no significant difference in cell morphology, attachment, or growth time between the ninth generation and the primary culture.

**Figure 2:**
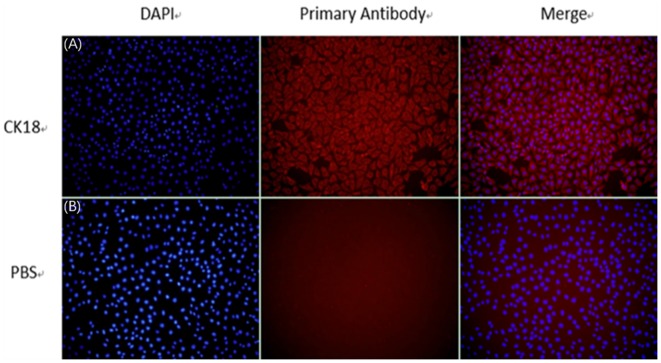
The identification of SEECs by CK18. **(A)** Cells incubating with CK18 and secondary antibody. **(B)** Cells incubating with PBS and secondary antibody (magnification ×200).

To determine whether the epithelial marker protein, CK18, can be expressed in established SEECs, an indirect immunofluorescence assay was conducted using mouse monoclonal antibody that targets CK18. The result revealed that the SEECs were reactive with CK18-specific antibody and that the negative group showed no red fluorescence (Figure 2). We concluded from this observation that CK18 can be efficiently expressed in the cells we purified, which strongly confirms that the established cells are epithelial cells.

### Growth Characteristics of SEECs

**Figure 3:**
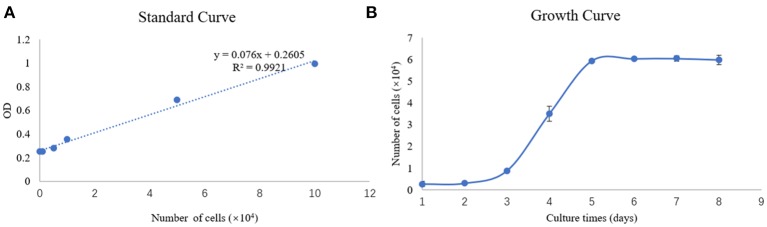
Standard curve and growth curve of SEECs. **(A)** Standard curve of SEECs. **(B)** Growth curve of SEECs.

To determine the growth properties of the newly established SEECs, the growth curve was carried out at passage 9 of the cell. The standard curve and growth curve of SEECs were shown in Figures 3A,B. In Figure 3A, the *R*^2^ of the standard curve is 0.9921. While in Figure 3B, SEECs grew slowly in the first 2 days and achieved the logarithmic phase and plateau phase at the third and fifth days, respectively.

### Result of Cell Viability Assay

**Figure 4:**
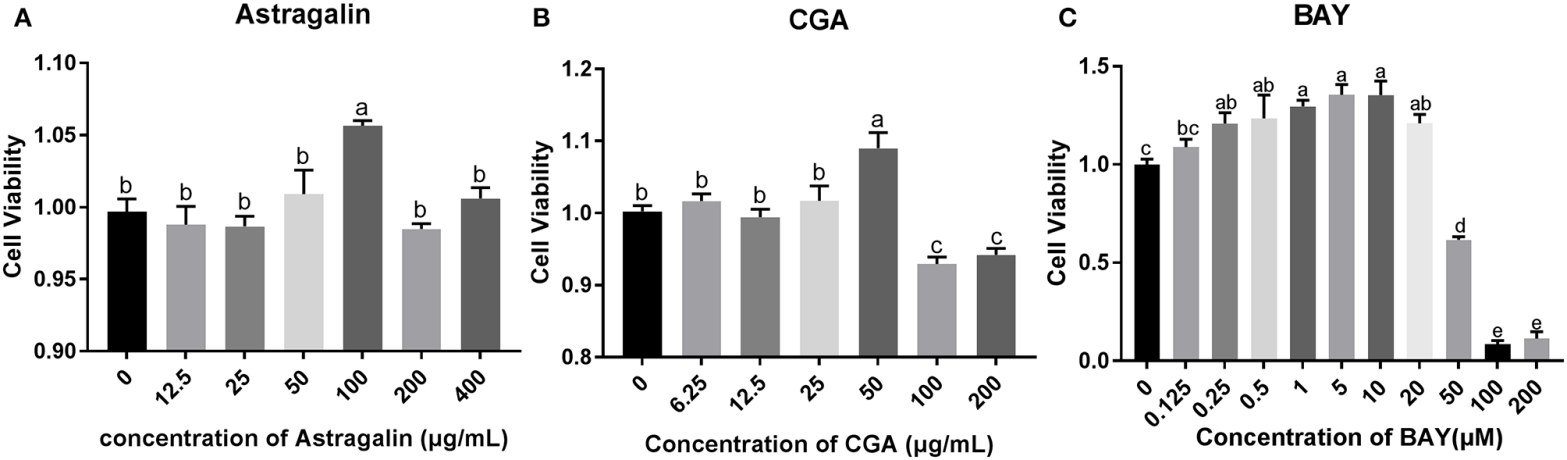
The cell viability assay result of astragalin, CGA, and BAY. **(A)** The cell viability assay result of incubating with astragalin for 24 h. **(B)** The cell viability assay result of incubating with CGA for 24 h. **(C)** The cell viability assay result of incubating with BAY for 24 h. Data are represented as the mean ± SD of three independent experiments with mixed SEECs from 15 sheep. Values with different lowercase letters were considered as statistically significant (*P* < 0.05). The accurate *P* values of this article were listed in Supplementary Tables 1–4.

To explore the appropriate concentration of the medicines in the present study, SEECs were treated with different doses of astragalin (12.5, 25, 50, 100, 200, and 400 μg/ml), CGA (6.25, 12.5, 25, 50, 100, and 200 μg/ml), and BAY (0.125, 0.25, 0.5, 1, 5, 10, 20, 50, 100, and 200 μM). In clinical treatment, the purpose of medication is not only to alleviate the symptoms of the disease but also to promote the recovery of the lesion, so that the drug effect will be better. Hence, the results indicate that the dose of 100 μg/ml of astragalin and 50 μg/ml of CGA significantly increased (*P* < 0.05) cell viability (Figure 4). As a result of this assay, we chose the dose of 100 μg/ml of astragalin, 50 μg/ml of CGA, and 5 μM of BAY to incubate SEECs to explore the effect of astragalin and CGA on *E. coli*-infected SEECs.

### Results of the *in vitro* Inflammation Model Establishment

**Figure 5:**
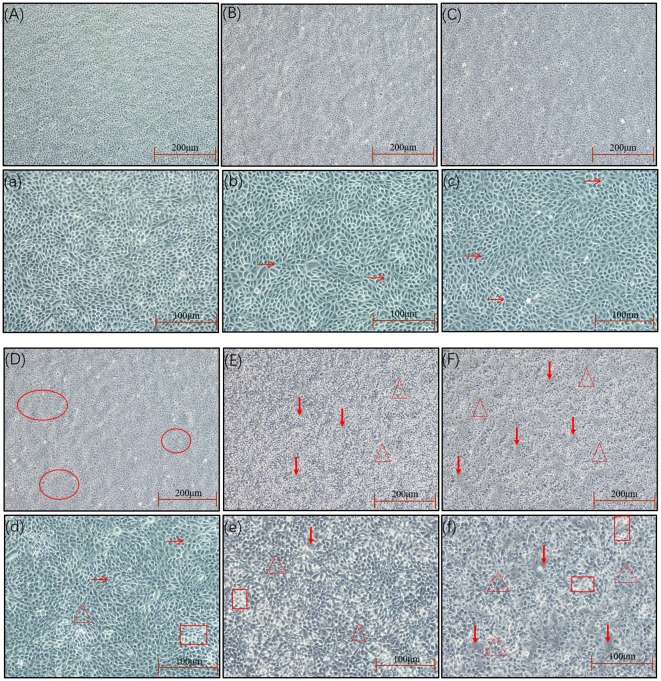
The cell morphology at different MOIs. The SEECs were non-treated or treated with five different MOIs of *E. coli* for 3 h, and cell morphologic changes were observed. **(A,a)** The cell morphology of normal SEECs. The cells were orbicular-ovate, with the edge clear and attached well. **(B,b)** The cell morphology at the MOI of 1:10. The cells showed no significant changes compared with normal SEECs, but with a few cell vacuolization (arrows). **(C,c)** The cell morphology at the MOI of 1:1. The cells showed no significant changes compared with normal SEECs, but the cells were not smooth and a few cell vacuolization (arrows) were observed. **(D,d)** The cell morphology at the MOI of 5:1. The cells in red circles in **D** become flattened with the edge blurring, and obvious cell vacuolization (arrows), transparence (rectangle), and cell shrinkage (triangle) were observed in **d**. **(E,e**) The cell morphology at the MOI of 10:1. The cells were apparently shrunken (triangle), transparent (rectangle), and detached (bold arrows). **(F,f)** The cell morphology at the MOI of 100:1. Abundant cell shrinkage (triangle), transparence (rectangle), and detachment (bold arrows) were observed.

**Figure 6:**
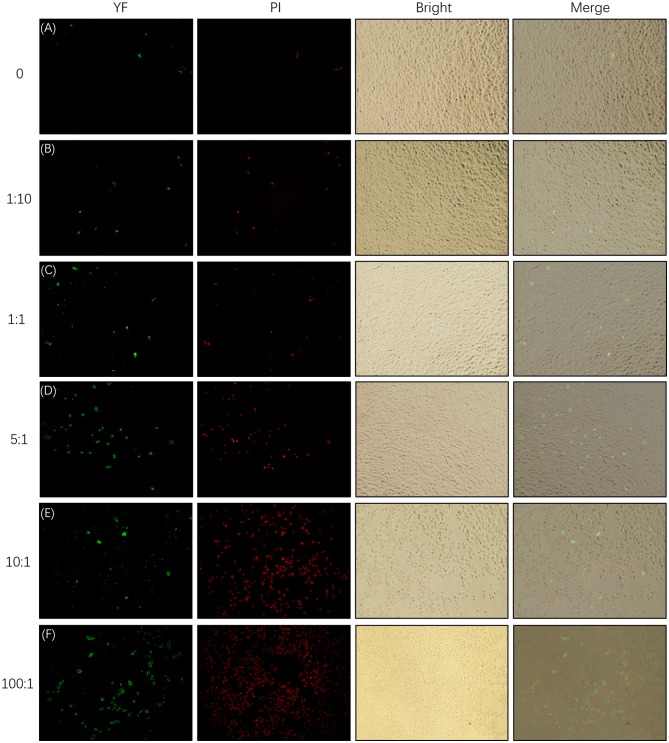
Apoptosis and necrosis of SEECs tested by YF-Annexin V and PI Apoptosis Kit. The SEECs were non-treated or treated with five different MOIs of *E. coli* for 3 h, followed by apoptosis and necrosis assays. Green fluorescence showed the apoptotic cells which were combined with YF-Annexin V, and red fluorescence showed the dead cells which were combined with PI. **(A)** Apoptosis and necrosis of SEECs in the non-infecting group. **(B)** Apoptosis and necrosis of SEECs infected by *E. coli* at the MOI of 1:10. **(C)** Apoptosis and necrosis of SEECs infected by *E. coli* at the MOI of 1:1. **(D)** Apoptosis and necrosis of SEECs infected by *E. coli* at the MOI of 5:1. **(E)** Apoptosis and necrosis of SEECs infected by *E. coli* at the MOI of 10:1. **(F)** Apoptosis and necrosis of SEECs infected by *E. coli* at the MOI of 100:1.

**Figure 7:**
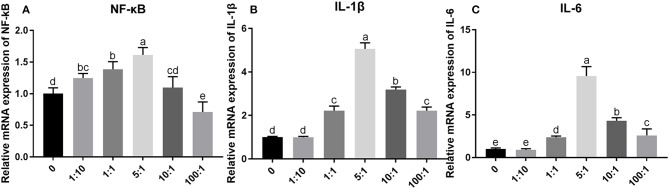
The relative mRNA expression of inflammatory cytokines induced by *E. coli* with different MOIs. The SEECs were non-treated or treated with five different MOIs of *E. coli* for 3 h, and the total RNA was isolated, followed by qPCR to detect the expression of NF-κB, IL-1β, and IL-6. **(A)** The relative mRNA expression of NF-κB induced by *E. coli* with different MOIs. **(B)** The relative mRNA expression of IL-1β induced by *E. coli* with different MOIs. **(C)** The relative mRNA expression of IL-6 induced by *E. coli* with different MOIs. Data are represented as the mean ± SD of three independent experiments with mixed SEECs from 15 sheep. Values with different lowercase letters were considered as statistically significant (*P* < 0.05).

The results of the *in vitro* inflammation model establishment were shown in Figures 5–**7**. As is shown in Figure 5, changes in cellular morphology gradually appeared as the MOI of *E. coli* increased, and significant changes of the cellular morphology were observed when the MOI of *E. coli* was 5:1, 10:1, and 100:1 where vacuolization, shrinkage, transparency, detachment, and even dead cells appeared.

As is shown in Figure 6, the apoptosis and necrosis of SEECs were increased with the MOI of *E. coli*, and numerous apoptotic and necrotic cells appeared when the MOI of *E. coli* was 5:1, 10:1, and 100:1. This result was consistent with the result of the morphological changes of SEECs.

The relative mRNA expression of NF-κB, IL-1β, and IL-6 increased with a dose-dependent effect when MOI was below 5:1, and it is decreased gradually when MOI exceeded 5:1 (Figure 7). The relative mRNA expression of NF-κB was significantly increased (*P* < 0.05) when compared with that in group C at MOIs of 1:1 and 5:1. The relative mRNA expression of IL-1β and IL-6 was significantly increased (*P* < 0.05) when compared with that in group C at MOIs of 1:1, 5:1, 10:1, and 100:1. These results indicate that the MOI of *E. coli* at 1:1 was appropriate to establish the inflammation model of SEECs.

### Astragalin and CGA Inhibit the mRNA Expression of Genes in the TLR4/NF-κB Signaling Pathway Induced by *E. coli* Infection

**Figure 8:**
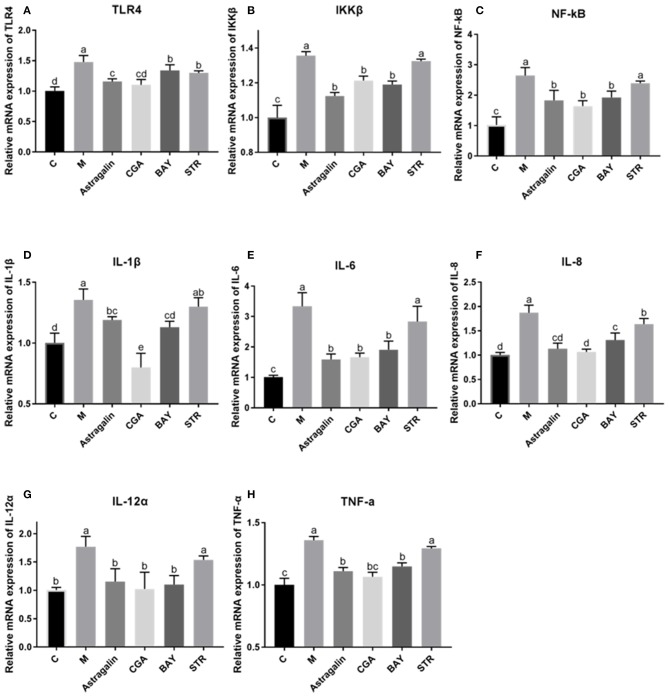
The relative mRNA expression of genes in the TLR4/NF-κB pathway. The SEECs were pretreated for 3 h with various compounds (astragalin, CGA, BAY, and STR) and then exposed to *E. coli* for 3 h, and the mRNA expressions of TLR4, IKKβ, NF-κB, IL-1β, IL-6, IL-8, IL-12α, and TNF-α were detected by qPCR. **(A)** The relative mRNA expression of TLR4. **(B)** The relative mRNA expression of IKKβ. **(C)** The relative mRNA expression of NF-κB. **(D)** The relative mRNA expression of IL-1β. **(E)** The relative mRNA expression of IL-6. **(F)** The relative mRNA expression of IL-8. **(G)** The relative mRNA expression of IL-12α. **(H)** The relative mRNA expression of TNF-α. Data are represented as the mean ± SD of three independent experiments with mixed SEECs from 15 sheep. Values with different lowercase letters were considered as statistically significant (*P* < 0.05).

As is shown in Figure 8, the mRNA expression of the genes (TLR4, IKKβ, NF-κB, IL-1β, IL-6, IL-8, IL-12α, and TNF-α) in the TLR4/NF-κB signaling pathway was significantly increased (*P* < 0.05) by about 45, 35, 160, 35, 230, 87, 77, and 36%, respectively, in group M compared with those in group C. Compared to the cells treated with *E. coli* only, the mRNA expression of IKKβ, NF-κB, IL-1β, IL-6, IL-8, IL-12α, and TNF-α was obviously reduced (*P* < 0.05) in cells pretreated with astragalin, CGA, and BAY for 3 h ahead of *E. coli*. But the mRNA expression of TLR4 in group BAY was significantly higher (*P* < 0.05) than that in groups C, astragalin, and CGA. Meanwhile, almost all the genes detected in group STR were increased (*P* < 0.05) compared with groups C, astragalin, and CGA.

### Astragalin and CGA Suppress the Relative Protein Expression of TLR4, pNF-κB, and pIκB Induced by *E. coli* Infection

**Figure 9:**
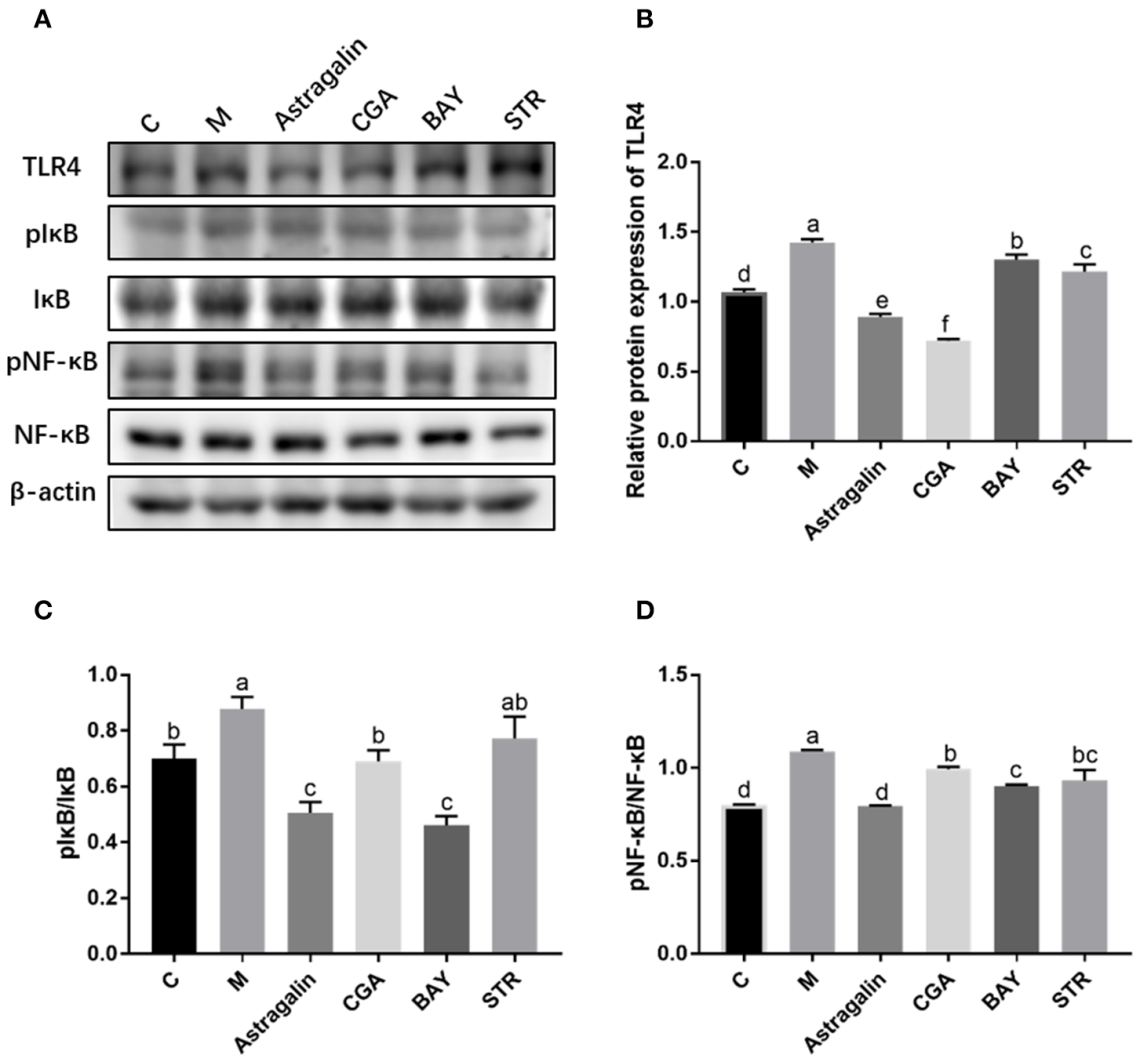
The relative protein expression of genes in the TLR4/NF-κB pathway. The SEECs were pretreated for 3 h with various medicines (astragalin, CGA, BAY, and STR) and then exposed to *E. coli* for 3 h, and the protein levels of TLR4, pIκB, IκB, pNF-κB, and NF-κB were determined by western blot analysis. **(A)** The protein bands of TLR4, pIκB, IκB, pNF-κB, NF-κB, and β-actin detected by western blot. **(B)** The IntDen of TLR4/β-actin. **(C)** The IntDen of pIκBα/IκBα. **(D)** The IntDen of pNF-κB/NF-κB. Data are represented as the mean ± SD of three independent experiments with mixed SEECs from 15 sheep. Values with different lowercase letters were considered as statistically significant (*P* < 0.05).

The protein bands of TLR4, pIκBα/IκBα, and pNF-κB/NF-κB, detected by western blot are shown in Figure 9A while the IntDens of TLR4/β-actin, pIκBα/IκBα, and pNF-κB/NF-κB are shown in Figures 9B–**D**, respectively. The expression of TLR4 in group M was significantly increased (*P* < 0.05) by about 34% compared with that in group C, and the expression of TLR4 in groups astragalin and CGA were significantly decreased (*P* < 0.05) compared with that in group M (Figure 9B). The expression of TLR4 in groups BAY and STR was significantly increased (*P* < 0.05) by about 23% and 21% compared with that in group C but was lower (*P* < 0.05) by about 8% and 15% than in group M. Also, the expression of TLR4 in groups BAY and STR was significantly higher (*P* < 0.05) than that in groups astragalin and CGA. The results in Figures 9C,D showed that the ratios of pIκBα/IκBα and pNF-κB/NF-κB in group M were greatly increased (*P* < 0.05) compared with that in group C by about 20 and 35%, respectively. Meanwhile, the ratios of pIκBα/IκBα and pNF-κB/NF-κB in groups astragalin, CGA, and BAY were obviously reduced (*P* < 0.05) compared with those in group M. Interestingly, the expression of TLR4 and the ratios of pIκBα/IκBα and pNF-κB/NF-κB in group astragalin were all remarkably decreased (*P* < 0.05) compared to group STR.

### Astragalin and CGA Reduce the Expression of the TLR4 and Inflammatory Cytokines Induced by *E. coli* Infection

**Figure 10:**
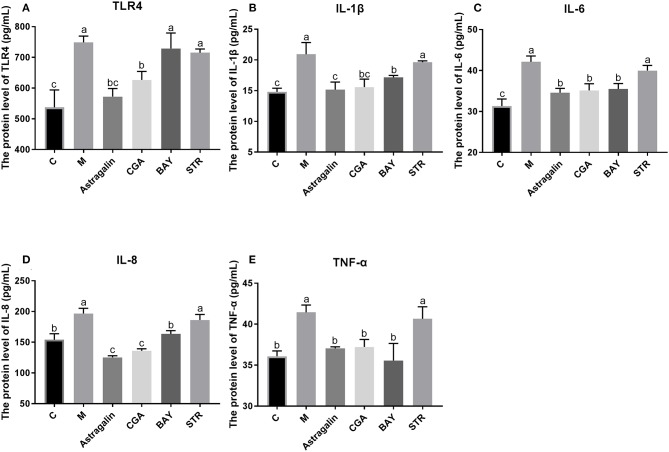
The protein expression of genes in the TLR4/NF-κB pathway. The SEECs were pretreated for 3 h with various medicines (astragalin, CGA, BAY, and STR) and then exposed to *E. coli* for 3 h, and the protein levels of TLR4, IL-1β, IL-6, IL-8, and TNF-α were determined by ELISA. **(A)** The protein expression of TLR4. **(B)** The protein expression of IL-1β. **(C)** The protein expression of IL-6. **(D)** The protein expression of IL-8. **(E)** The protein expression of TNF-α. Data are represented as the mean ± SD of three independent experiments with mixed SEECs from 15 sheep. Values with different lowercase letters were considered as statistically significant (*P* < 0.05).

As is shown in Figure 10, the protein expression of TLR4 and inflammatory cytokines (IL-1β, IL-6, IL-8, and TNF-α) in the TLR4/NF-κB signaling pathway was significantly increased (*P* < 0.05) by about 39, 40, 35, 27, and 14%, respectively, in group M compared with those in group C. Compared with that in group M, the protein expression of the genes was obviously reduced (*P* < 0.05) when cells were pretreated with astragalin and CGA for 3 h. The protein expression of IL-1β, IL-6, IL-8, and TNF-α in group BAY was significantly decreased (*P* < 0.05) when compared with group M, while the protein expression of TLR4 showed no significant difference (*P* > 0.05). Meanwhile, the protein levels of TLR4, IL-1β, IL-6, IL-8, and TNF-α in group STR also showed no significant difference (*P* > 0.05) compared with those in group M but were significantly higher (*P* < 0.05) than those in group C.

## Discussion

Endometritis is characterized by inflammation of the endometrium and has seriously hindered the development of the breeding industry (19). Although the clinical application of antibiotics in the treatment of endometritis has some efficacy, it has led to the emergence of drug-resistant strains (20). Bacteria are the main cause of endometritis, and one of the most common bacteria is *E. coli* (21, 22). It has been reported that 15–20% of dairy cow endometritis is involved with bacterial infection, indicating that preventing and treating endometritis induced by *E. coli* are of great significance (23). TCM including compound and monomer keeps getting more attention due to its multiple biological activities, such as anti-inflammation, antioxidant, and immunity enhancement, especially its non-tolerance to bacteria and virus (24, 25). A lot of studies showed that TCM was effective in preventing organisms from a bacterial infection (26). For these reasons, many researchers are focused on the positive effect of TCM on preventing and treating endometritis of dairy cows, but few studies aimed at the preventive and therapeutic effects of TCM on sheep endometritis. In this study, we isolated and purified SEECs and created an *in vitro* model of sheep endometritis via infecting SEECs with *E. coli* to explore the protective effect of astragalin and CGA and its possible protective mechanisms. The results showed that astragalin and CGA could inhibit the progress of the inflammatory response of SEECs induced by *E. coli* infection via a mechanism related to the inhibition of the TLR4/NF-κB signaling pathway.

*E. coli* is a gram-negative bacterium, which contains the special component LPS in the cytoderm. LPS induces inflammatory response via activating the TLR4/NF-κB signaling pathway (27). Mammalian TLR4 is the signal-transducing receptor activated by the bacterial LPS, and LPS first binds to the cluster of differentiation-14 (CD14) receptor, which then transfers them to TLR4. In the MyD88-dependent pathway, TLR4 combines with MyD88 via TIR after being activated by LPS. MyD88 recruits IRAK4, thereby allowing the association of IRAK1. Then, IKK is activated after a series of complicated biochemical reactions. On the other hand, Tir domain-containing adaptor inducing interferon-beta (TRIF) and Trif-related adaptor molecule (TRAM) link TLR4 to pathways that lead to the activation of TNF receptor-associated factor 6 (TRAF6) in the MyD88-independent pathway, followed by the activation of IKK. In both the MyD88-dependent and MyD88-independent pathways, the NF-κB, which was inhibited by combining with IκB, is released following the activated IKK phosphorylating IκB. Afterward, the activated NF-κB transfers into the nucleus after phosphorylation binds to specific sites and initiates the transcription of pro-inflammatory factors, including IL-1β, IL-6, IL-10, IL-12, and necrocytosis TNF-α (8, 9, 28, 29). However, *E. coli* infection at an improper MOI will induce obvious apoptosis (30, 31). Therefore, the *in vitro* inflammation model of SEECs in this study was created by infecting SEECs with *E. coli*, and the cellular morphology, apoptosis and necrocytosis, and the mRNA expression of NF-κB, IL-1β, and IL-6 were used as the evaluation criteria to choose the proper MOI of *E. coli*. As a result, the expressions of NF-κB, IL-1β, and IL-6 in SEECs were increased after infection with *E. coli*, and there was a dose-dependent effect when the MOI was beneath 5:1. But when the MOI of *E. coli* achieved 5:1, 10:1, and 100:1, the cell morphology changed remarkably. For instance, the cell became flattened, the edge blurred, and evident cell vacuolization, transparence, cell shrinkage, and detachment were observed. Meanwhile, numerous apoptotic and necrotic cells appeared when the MOI of *E. coli* was 5:1, 10:1, and 100:1. In conclusion, when the MOI of *E. coli* achieved 1:1, it induced an inflammatory response without numerous apoptosis and necrocytosis. This result was consistent with the conclusion of a previous study which revealed that an appropriate MOI of *E. coli* could significantly induce inflammatory response rather than cell death (32).

**Figure 11:**
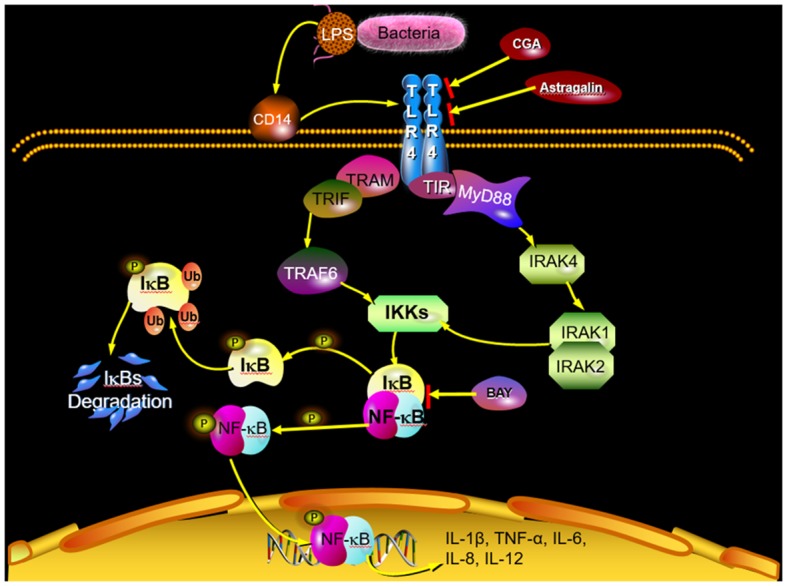
Scheme summarizing the mechanism of the protective effect of astragalin and CGA on *E. coli*-induced endometritis. *E. coli* infection activates the TLR4/NF-κB signaling pathway. Activated NF-κB can be translocated from the cytosol to the nucleus, where it activates the transcription of genes that include IL-1β, IL-6, IL-8, IL-12, and TNF-α. Astragalin and CGA could inhibit the inflammatory response by blocking TLR4 activation.

With the development of TCM, many researchers focus on the mechanism of different natural herbs and different active ingredients including flavonoids (33), phenols (34), alkaloids (35), and polysaccharides (36). Deng et al. (37) have demonstrated that inhibiting the progress of inflammation could effectively alleviate the injury to the animals induced by endometritis. Jiang et al. (20) suggested that natural herbs have remarkable curative effects in the treatment of endometritis via inhibiting the progress of inflammation. It is reported that treatment with astragalin suppressed inflammatory response by regulating NF-κB and mitogen-activated protein kinase (MAPK) signaling pathways in leptospirosis-infected uterine and endometrium epithelial cells of mice (38). Another study also revealed that astragalin exerts anti-inflammatory properties possibly via the inactivation of TLR4-mediated NF-κB and MAPK signaling pathways in LPS-stimulated mMECs (39). In addition, some researchers demonstrated the hepatoprotective nature of CGA by attenuating the pro-inflammatory and apoptotic mediators and improving antioxidant competence in hepatic tissues (40). In the preliminary experiment, we have revealed that astragalin and CGA showed no observable antibacterial activity at the concentrations of 100 and 50 μg/ml, respectively (Supplementary Figure 2) and induced no significant changes (*P* > 0.05) of inflammatory response compared with untreated cells (Supplementary Figure 3). And in the present study, *E. coli* infection induced inflammatory response via activating the TLR4/NF-κB signaling pathway, and astragalin and CGA inhibited the inflammatory progress by means of reducing the expression of TLR4 (Figure 11). This mechanism was different from the anti-inflammation effect of BAY, which inhibited the inflammatory progress by inhibiting the activation of IκB and NF-κB. However, whether there were other genes in TLR4/NF-κB involved with the anti-inflammation effect of astragalin and CGA still needs further research. Furthermore, although STR could inhibit the *E. coli* proliferation, it could not alleviate the inflammatory progress at 3 h after *E. coli* infection.

In summary, our data demonstrated that astragalin (100 μg/ml) and CGA (50 μg/ml) possessed anti-inflammatory effects in *E. coli*-induced inflammation of SEECs by inhibiting the TLR4-mediated activation of the NF-κB signaling pathway. And the proper mechanism was blocking the activation of TLR4. Accordingly, these findings demonstrated that astragalin and CGA can serve as anti-inflammatory agents combined with antibacterial agents in sheep endometritis induced by *E. coli* infection.

## Data Availability Statement

All datasets generated for this study are included in the article/Supplementary Material.

## Author Contributions

XH and SY came up with and designed the study. XH, JH, XZ, and TZ performed the experiments. XH, HH, and QL performed the statistical analysis. XH, YX, RN, and MW wrote the manuscript. YP, RN, and YC revised the manuscript.

## Conflict of Interest

The authors declare that the research was conducted in the absence of any commercial or financial relationships that could be construed as a potential conflict of interest.
