# Supplementary material for: Anti-inflammatory Effect of Astragalin and Chlorogenic Acid on Escherichia coli-Induced Inflammation of Sheep Endometrial Epithelium Cells
Source: Front Vet Sci. 2020 Apr 30;7:201. doi: 10.3389/fvets.2020.00201 (PMC7205036; doi:10.3389/fvets.2020.00201)
Supplement: Supplementary file 1 [file Data_Sheet_1.docx]

Supplementary Material

# Supplementary procedure of ELISA

ELISA procedure was as follows and supplied as supplementary materials.

(1) Add standard: Set Standard wells, testing sample wells. Add standard 50μl to standard well.

(2) Add sample: Set blank wells separately (blank comparison wells don’t add sample and HRP-Conjugate reagent, other each step operation is same). Testing sample well. Add Sample dilution 40μl to testing sample well, then add testing sample 10μl (sample final dilution is 5-fold), add sample to wells, don’t touch the well wall as far as possible, and gently mix.

(3) Add enzyme: Add HRP-Conjugate reagent 100μl to each well, except blank well.

(4) Incubate: After closing plate with Closure plate membrane, incubate for 60 min at 37℃.

(5) Configurate liquid: 20-fold wash solution diluted 20-fold with distilled water and reserve.

(6) Washing：Uncover Closure plate membrane, discard liquid, dry by swing, add washing buffer to every well, still for 30s then drain, repeat five times, dry by pat.

(7) Color: Add Chromogen Solution A 50ul and Chromogen Solution B to each well, evade the light preservation for 15 min at 37℃

(8) Stop the reaction: Add Stop Solution 50μl to each well, Stop the reaction (the blue color change to yellow color).

(9) Assay: take blank well as zero, Read absorbance at 450nm after Adding Stop Solution and within 15min.

# Supplementary Figures

2.1 The chemical structure of Astragalin and CGA were shown in Supplementary Figure 1.


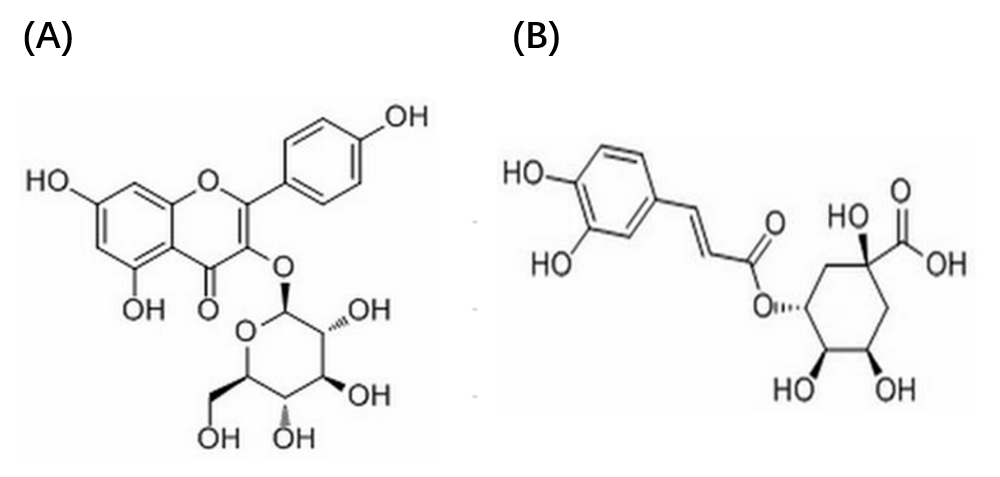


**Supplementary Figure 1.** Chemical structure of Astragalin and CGA. (A) Chemical structure of Astragalin; (B) Chemical structure of CGA.

2.2 The in vitro antibacterial experiment of Astragalin and CGA

The Astragalin and CGA were dissolved with dimethylsulfoxide (DMSO) and diluted with saline to the concentration of 400, 200, 100, 50 and 25 μg/mL. 100 μL Astragalin and CGA of each concentration were added into a 96-well plate, and 100 μL E. coli solution of 10^7^ CFU/mL were added in each well. After incubating at 37℃ for 24 hr, 40 μL iodinitrotetrazolviolet (INT) was added to each well and incubated at 37℃ for 30 min. In addition, the positive and negative control were using STR and saline to replace the compound. The compound showed no antibacterial activity if the solution becomes cloudy with pink precipitation, while the compound showed observable antibacterial activity if the solution is clear. The experiment was repeated three times.

The result of the in vitro antibacterial activity of Astragalin and CGA were shown in Supplementary Figure 2. The solution in wells incubated with STR were clear and the solution in wells incubated with saline become cloudy with pink precipitation. The solution which incubated with Astragalin (100 μg/mL) and CGA (50 μg/mL) were turbid with pink precipitation. These results showed Astragalin and CGA at the concentration of 100 μg/mL and 50 μg/mL respectively showed no observable antibacterial activity.


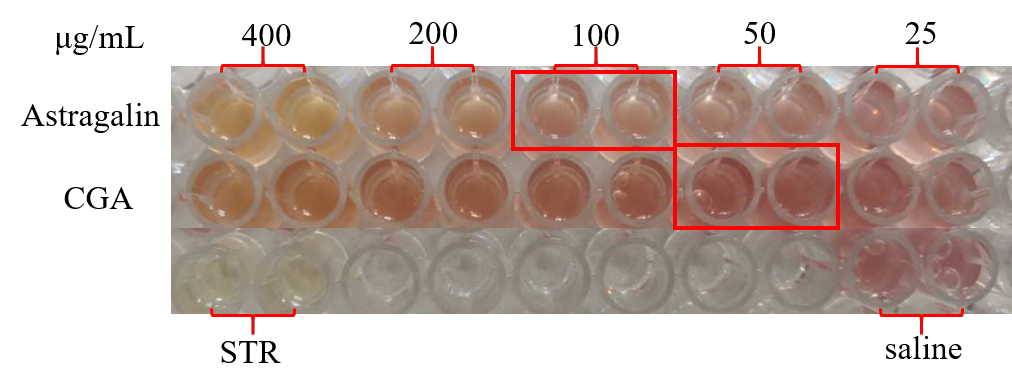


**Supplementary Figure 2.** The antibacterial experiment in vitro of Astragalin and CGA. In the first row, various concentrations of Astragalin were exposure E. coli; In the second row, various concentrations of CGA were exposure E. coli; In the third row, the STR and saline were exposure to E. coli.

2.3 The effect of Astragalin and CGA on the inflammatory cytokines expression in SEECs

To evaluate the effect of Astragalin and CGA on the inflammatory cytokines expression in SEECs, cells were seeded into a 6-well plate for 24 hr and exposure to Astragalin or CGA at 37℃ for 6 hr, then the cells and the supernatant were collected to detect the expression of IL1β, IL6 and TNF-α by ELISA and qPCR.

The result were showed in supplementary figure 3, the mRNA and protein expression of IL1β, IL6 and TNF-α in both cells treated with Astragalin or CGA showed no significant difference compared with untreated cells, indicating that Astragalin and CGA don’t induce inflammatory response on SEECs.


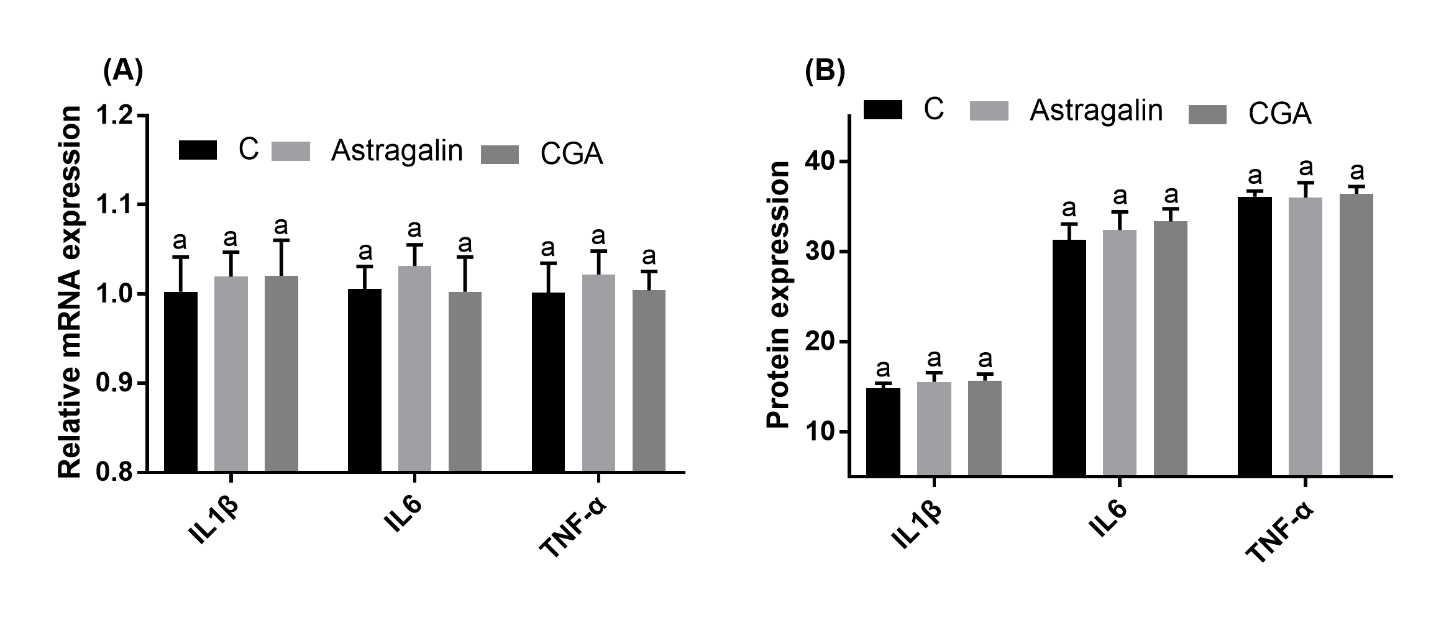


**Supplementary Figure 3.** The effect of Astragalin and CGA on the inflammatory cytokines expression in SEECs. Cells were exposure to Astragalin or CGA at 37℃ for 6 hr, and the cells and the supernatant were collected to detect the expression of IL1β, IL6 and TNF-α by qPCR and ELISA. (A) The effect of Astragalin and CGA on the relative mRNA expression of inflammatory cytokines in SEECs; (B) The effect of Astragalin and CGA on the protein expression of inflammatory cytokines in SEECs. Data are represented as the mean ± SD of three independent experiments. Different lowercase letters were considered as statistically significant (*P*<0.05).

# Supplementary Tables

3.1 The actual P values of each comparison in qPCR assay for the establishment of in vitro inflammation model were shown in Supplementary Table 1.

Supplementary Table 1. The actual P values of each comparison in qPCR assay for the establishment of in vitro inflammation model

3.2 The actual P values of each comparison in qPCR assay for evaluating the anti-inflammatory effect of Astragalin and CGA were shown in Supplementary Table 2.

Supplementary Table 2. The actual P values of each comparison in qPCR assay for evaluating the anti-inflammatory effect of Astragalin and CGA

3.3 The actual P values of each comparison in WB assay were shown in Supplementary Table 3.

Supplementary Table 3. The actual P values of each comparison in WB assay

3.4 The actual P values of each comparison in ELISA were shown in Supplementary Table 4.

Supplementary Table 4. The actual P values of each comparison in ELISA
